# The Role of NF-κB and H3K27me3 Demethylase, Jmjd3, on the Anthrax Lethal Toxin Tolerance of RAW 264.7 Cells

**DOI:** 10.1371/journal.pone.0009913

**Published:** 2010-03-29

**Authors:** Nando Dulal Das, Kyoung Hwa Jung, Young Gyu Chai

**Affiliations:** Division of Molecular and Life Science, Hanyang University, Ansan, Korea; Charité-Universitätsmedizin Berlin, Germany

## Abstract

**Background:**

In *Bacillus anthracis*, lethal toxin (LeTx) is a critical virulence factor that causes immune suppression and toxic shock in the infected host. NF-κB is a key mediator of the inflammatory response and is crucial for the plasticity of first level immune cells such as macrophages, monocytes and neutrophils. In macrophages, this inflammatory response, mediated by NF-κB, can regulate host defense against invading pathogens. A Jumonji C family histone 3 lysine-27 (H3K27) demethylase, Jmjd3, plays a crucial role in macrophage plasticity and inflammation. Here we report that NF-κB and Jmjd3 can modulate the LeTx intoxication resistance of RAW 264.7 cells.

**Principal Findings:**

This study showed that a 2 h exposure of macrophages to LeTx caused substantial cell death with a survival rate of around 40%. The expression of the Jmjd3 gene was induced 8-fold in intoxication-resistant cells generated by treatment with lipopolysaccharides of RAW 264.7 cells. These intoxication-resistant cell lines (PLx intox and PLxL intox) were maintained for 8 passages and had a survival rate of around 100% on secondary exposure to LeTx and lipopolysaccharides. Analysis of NF-κB gene expression showed that the expression of *p100, p50* and *p65* was induced around 20, 7 and 4 fold, respectively, in both of the intoxication-resistant cell lines following a 2 h treatment with PLxL (0.1+0.1+1 µg/ml). In contrast, these NF-κB genes were not induced following treatment with PLx treatment at the same concentrations.

**Conclusions:**

Although LeTx influences macrophage physiology and causes defects of some key signaling pathways such as GSK3β which contributes to cytotoxicity, these results indicate that modulation of NF-κB by p50, p100 and Jmjd3 could be vital for the recovery of murine macrophages from exposure to the anthrax lethal toxin.

## Introduction

The transcription factor, NF-κB, regulates numerous genes involved in cell survival (cellular inhibitor of apoptosis; *c-IAP*, cellular FLICE-like inhibitory protein; *c-FLIP* and B-cell lymphoma extra large, *Bcl-xl*), proliferation (*TNF, IL-1, IL-6, cyclin-D1* and *c-myc*), angiogenesis (*VEGF*, *TNF, IL-1* and *IL-8*), inflammation (*TNF, IL-1* and chemokines), invasion (cyclooxygenase-2; *COX-2* and matrix metalloprotease 9; *MMP9*) and metastasis (intercellular adhesion molecule; *ICAM-1* and vascular cell adhesion molecule; *VCAM-1*) [1]. Although the function of NF-κB is primarily mediated by a single abundant species, the p65-p50 heterodimer, there are five transcription factors of the mammalian NF-κB family: p65 (also called RelA), c-Rel, RelB, p50 and p52. Depending on the cell status, these proteins act through the formation of homo- or heterodimers. Proteins of the NF-κB contain a highly homologous Rel region that mediates protein-DNA interactions with its N-terminal domain and dimerization with its C-terminal domain. Depending on the cell type and the differentiation status, the relative abundance of each dimer may vary [1], [2].

The present study focuses on the epigenetic role of NF-κB on cell survival following lethal toxin (LeTx) shock. LeTx is a binary toxin of *Bacillis anthracis* composed of protective antigen (P), a molecular transporter allowing receptor-mediated entry and release of Lx into the cytosol and lethal factor (Lx), a zinc metalloprotease that cleaves the N-terminal end of mitogen-activated protein kinase (MAPK) kinases (MEK) 1 to 7, with the exception of MEK5, resulting in the inactivation of most of their downstream signaling cascades. P binds to either of two surface receptors: anthrax receptor 1 (also known as the tumor endothelial marker 8) and anthrax receptor 2 (also known as the capillary morphogenesis gene-2). While anthrax receptor 2 is widely distributed in human tissues, anthrax receptor 1 is expressed abundantly in macrophages and is also found in other cells, including endothelial cells and several tumor cells [3], [4], [5], [6]. It has been reported that NACHT-leucine-rich repeat and pyrin domain-containing protein 1b (NALP1b) in mice acts as the host factor that confers rapid LeTx cytotoxicity [7]. However, human macrophages lacking NALP1b are resistant to rapid necrotic cell death induced by LeTx. Instead, LeTx was shown to cause delayed apoptotic cell death of differentiated macrophages and inhibit cell proliferation and differentiation, most likely mediated through MAPK inhibition [8], [9], [10].

The exact mechanism of anthrax pathogenesis by the inhibition of cell proliferation by LeTx remains obscure. Interestingly, recovery from prolonged MEK-cleaving Lx activity required cell proliferation, which was mediated in some cells through an adaptive response by the induction of the phosphatidylinositol 3-kinase (PI3K)/Akt/GSK3 signaling pathway. This suggests that the recovery from cellular LeTx toxicity somehow depends either on the activation of PI3K/Akt pathway or on protection from cell cycle arrest by the GSK3-inhibitor [11]. Although impaired immune response, cell lysis due to loss of ions and degradation of survival factors are critical components of LeTx-induced cell death [12], [13], in macrophages, a short exposure to LeTx primarily down regulates NF-κB and GSK3 regulated genes [14], [15]. Several kinases, including PI3K/Akt kinase, signal through NF-κB for cell survival. In non-stimulated cells, the basal nuclear NF-κB levels may regulate the expression of certain genes required for cell survival [16]. However, NF-κB can regulate the induction of Jmjd3 which is responsible for macrophage plasticity and differentiation, by binding to the to the three kB sites on its promoter. Jmjd3, an active hydroxylase and H3K27me3 demethylase, is quickly and strongly induced in macrophages exposed to bacterial products and inflammatory cytokines [17]. Since, NF-κB acts broadly to influence gene expression events that impact cell survival, differentiation, and proliferation [18], the present study examines the possibility that differential induction of NF-κB in the cells that have developed resistance to LeTx intoxication may be mediated via the formation of open chromatin by Jmjd3.

## Results

### The effect of LeTx and LPS on the RAW 264.7 cells

**Figure 1:**
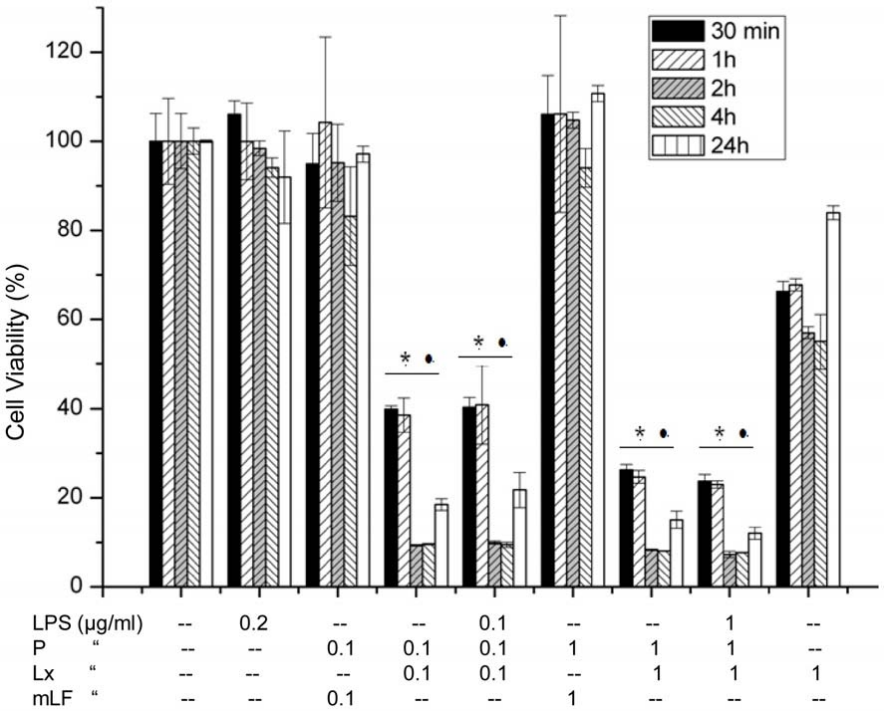
Cell viability assay. RAW 264.7 cells were treated with different combinations of P, Lx, LPS and mLF for 30 min to 24 h. After 24 hours, cells treated with both high and low concentrations of PLx and PLxL showed significantly less survival than untreated cells. Treatment with 0.2 µg/ml LPS and mLF had a negligible effect on cell viability whereas Lx with P and LPS treatment resulted in severe cell death except only Lx (1 µg/ml). Data shown are representative of three independent experiments, * •*P*<0.05, compared with untreated and PmLF treated cells respectively. Values represent mean ± SD (N = 3).

The anthrax toxin, LeTx, is specifically cytotoxic to macrophages, causing apoptosis of human macrophages [8]. Down regulation of the kinesin motor protein Kif1C resulted in the resistance of some murine macrophages to LeTx induced cytotoxicity, whereas up-regulation of this protein increased the sensitivity of cells to LeTx [15]. The effect of LeTx, P, Lx and mutant lethal factor (mLF), alone and in combination, on RAW 264.7 cells was investigated in incubations from 30 min to 24 h in duration. Two different combinations of LeTx and LPS concentration resulted in significant cell death even within 30 min. LeTx/PLx (0.1+0.1 & 1+1 µg/ml) and PLxL (0.1+0.1+0.1 & 1+1+1 µg/ml) greatly decreased absorbance in cell proliferation assays at both lower and higher concentrations compared to untreated cell controls (Fig. 1). Treatment with 0.2 µg/ml LPS and mLF with P had a negligible effect on cell viability, and only Lx (1 µg/ml) did not result in severe cell death (Fig. 1).

### Induction of Jmjd3 by LPS and with LeTx and LPS (PLxL)

**Figure 2:**
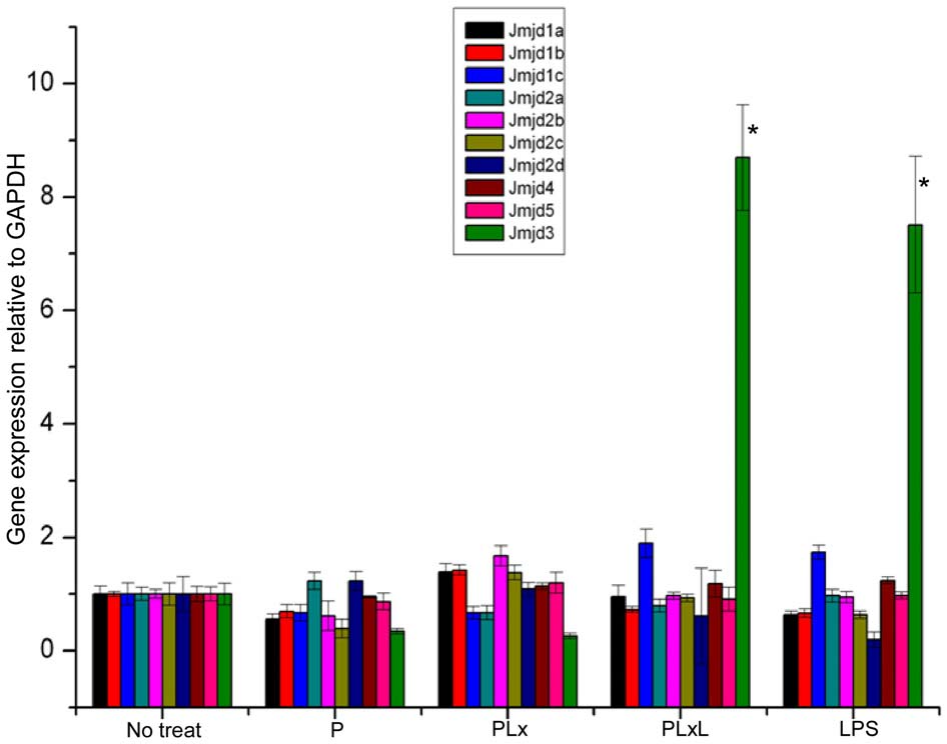
Induction of Jmjd3 by LPS alone and in combination with P, Lx and LPS (PLxL). Expression of different JmjC genes in Raw 264.7 macrophages stimulated with P (0.1 µg/ml), PLx (0.1+0.1 µg/ml), PLxL (0.1+0.1+1 µg/ml) or LPS (1 µg/ml) for 2 h. *Jmjd3* was significantly up-regulated (>8 fold) only in cells treated with PLxL and LPS than No treated cells (*P<0.05). Data shown are representative of three independent experiments. Results are expressed as the mean ± SD of triplicate wells.

Macrophages and their progenitors also show some degree of plasticity when exposed to inflammatory stimuli. It was reported that histone H3 lysine 9 methylation (H3K9me) was erased from the promoters of some slowly activated inflammatory genes upon activation [19]. The requirement of NF-κB for inflammatory gene expression is supported by recent evidence that showed that Jmjd3 induction by LPS was strongly inhibited by IκBα-SR [17]. Analysis of different upstream regions of Jmjd3 showed the presence of three κB sites. A ChIP experiment with an antibody directed against the NF-κB subunit p65/RelA revealed the presence of p65/RelA in LPS-stimulated cells at region 2, which lies upstream of the second exon. Therefore, inducible transcription of Jmjd3 depends on functional κB sites [17]. In this study, gene expression of JmjC following different combinations of treatments, including P, PLx, PLxL and LPS, was analyzed. PLx and PLxL treatment resulted in cell death in 30 to 40% of cells. A cell lysate of these cells was collected after washing. After 2 h stimulation with PLxL and LPS, only Jmjd3 was significantly up-regulated (>8 fold) in Raw264.7 cells compared to untreated cell controls (Fig. 2).

### Establishment of LeTx intoxication-resistant cells

**Figure 3:**
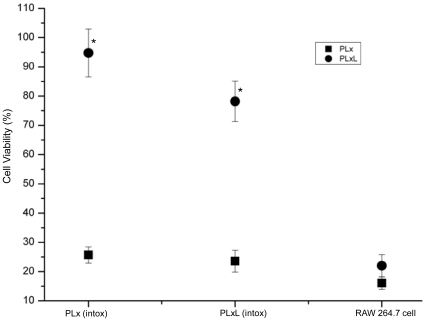
Cell viability assay for intoxication-resistant cells. Both PLx and PLxL intoxication-resistant cells were treated for 2 h with PLx (0.1+0.1 µg/ml) or PLxL (0.1+0.1+1 µg/ml). The survival rate for both cell lines treated with PLxL was around 100% whereas it was only around 20% for PLx treatment. RAW 264.7 cells had a very low survival rate for both PLx and PLxL treatment. Data shown are representative of two independent experiments, *P<0.05 compared with PLxL treated RAW 264.7 cells. Values represent mean ± SD (N = 3).

An initial cell proliferation assay showed that both lower and higher concentrations of PLx (0.1+0.1 & 1+1 µg/ml) and PLxL ((0.1+0.1+0.1 & 1+1+1 µg/ml)) were cytotoxic to cells within 30 min and that they had higher cytotoxicity at 2 h and 4 h. These data are compatible with a previous study in which more than 80% of cells were lysed within 4 h [20]. This study also observed that the remaining 20% cells showed resistance to LeTx [20]. To create a population of cells resistant to LeTx intoxication, RAW 264.7 cells were treated with levels of both PLx and PLxL that resulted in 50% cell death after 24 hours. The remaining cells were washed to remove dead cells and cultured for a further 8 passages (Table 1). The two intoxication-resistant cell lines were termed “PLx intox” and “PLxL intox”. Cell proliferation assays of the initial, 4^th^ and 8^th^ passages revealed significantly less cytotoxicity after stimulation with LPS (data not shown). Interestingly, treatment of RAW 264.7 cells with PLx and PLxL resulted in a cell survival rate of only 20% whereas almost 100% of the intoxication-resistant cells survived (Fig. 3). These results suggest that LPS stimulation is likely responsible for the survival of intoxication-resistant cells and might be necessary for the induction of NF-κB regulated genes along with Jmjd3.

**Table 1:** Establishment of intoxication-resistant cells.

Treatment Pattern, for 24 h	Steps
P (0.1 µg/ml)	1. Washing and let the cell grow for overnight and collection of total RNA
PLx (0.1+0.1 µg/ml)	2. Maintaining the treated cell up to 8^th^ passages
PLxL (0.1+0.1+1 µg/ml)	
LPS (1 µg/ml)	

PLx and PLxL treated cells called PLx and PLxL intoxication (intox) resistant cells respectively.

### Induction of *Jmjd3* in intoxication-resistant cells

**Figure 4:**
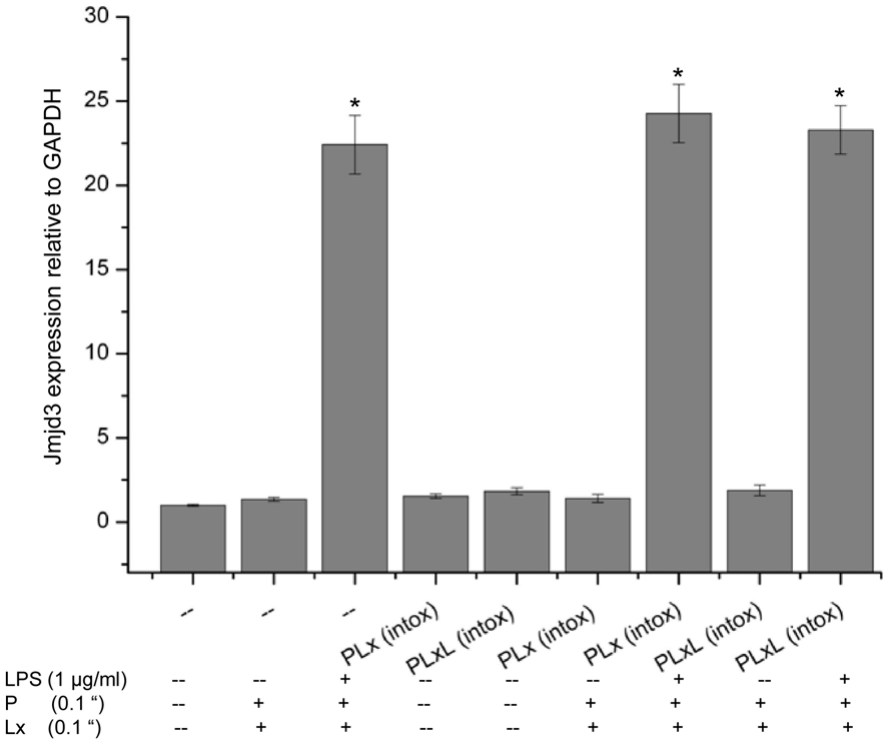
Induction of *Jmjd3* expression in intoxication-resistant (PLx and PLxL) and RAW 264.7 cells. Treatment of RAW 264.7 and intoxication-resistant cells with PLxL (0.1+0.1+1 µg/ml) showed almost identical *Jmjd3* induction than without treatment. Treatment of cells with PLx (0.1+0.1 µg/ml) did not result in any significant change. Data shown are representative of two independent experiments, *P<0.05 compared with untreated RAW 264.7 cells. Results are expressed as the mean ± SD of triplicate wells.

Histone modification plays a vital role in the induction of inflammatory gene expression. The covalent modifications of histones play a major role in gene regulation by affecting chromatin compaction and thereby DNA accessibility. These changes to histones at various positions result in either transcriptional repression (such as H3K9 me3 and H3K27me3 methylation) or in activation (H3K4me3, H3-Serine10 phosphorylation and H3K9 acetylation) [21], [22]. The expression levels of Jmjd3, also necessary for gene activation, were compared in intoxication-resistant cells versus Raw 264.7 cells treated with various molecules. Interestingly, after a 2 h treatment with PLxL (0.1+0.1+1 µg/ml), *Jmjd3* induction was similar in both in Raw 264.7 and intoxication-resistant cells while treatment with PLx (0.1+0.1 µg/ml) did not affect *Jmjd3* induction (Fig. 4).

### Induction of NF-κB genes in intoxication-resistant cells

**Figure 5:**
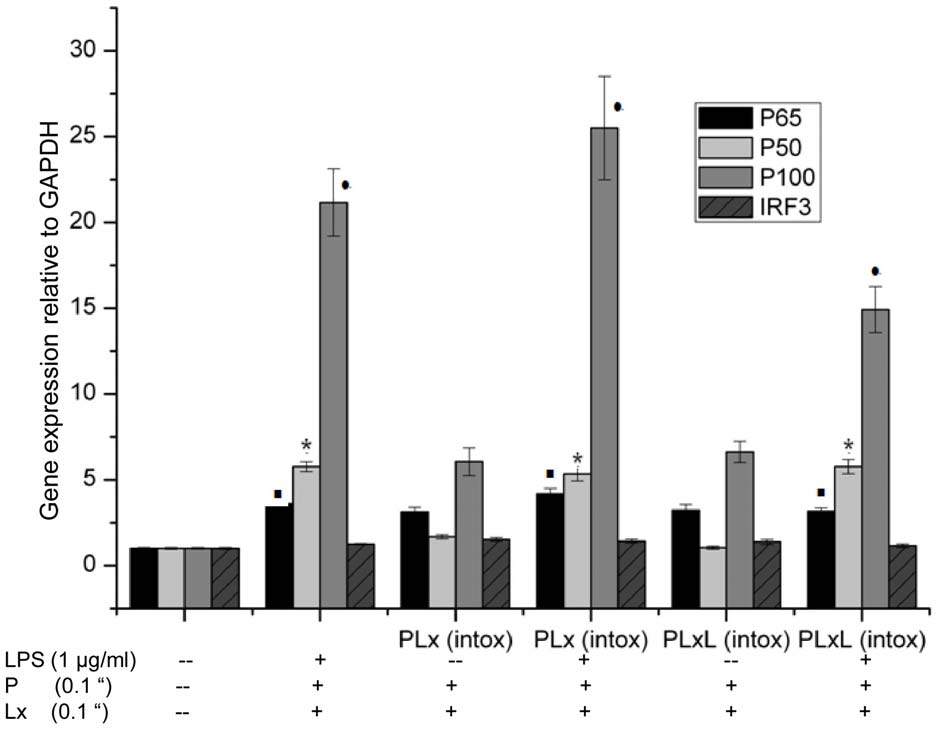
Induction of NF-κB genes in intoxication-resistant (PLx and PLxL) and RAW 264.7 cells. Treatment of Raw 264.7 and intoxication-resistant cells with PLxL (0.1+0.1+1 µg/ml) resulted in a 20, 7 and 4 fold induction of *p100, p50* and *p65*, respectively (▪*•*P*<0.05, compared with respective expressions in untreated RAW 264.7 cells). There was also a small induction of *IRF3* in intoxication resistant cells. Data shown are representative of two independent experiments. Results are expressed as the mean ± SD of triplicate wells.

Different NF-κB gene products can form homo- and heterodimers depending on cell type and gene regulation. While p65–p50 heterodimers are predominant, some homodimers, such as p50 and p52, act as repressors because these proteins lack the transcription activation domain present in RelA, RelB, v-Rel and c-Rel [23], [24]. The p50 homodimer is abundant in endotoxin-tolerant macrophages [25] as well as in tumor-associated macrophages [26], [27]. The pattern of expression of NF-κB signaling genes in RAW 264.7 cells after 60 min, 90 min and 180 min of LeTx exposure was determined by microarray analysis (data not shown). The microarray datasets were analyzed using Ingenuity pathway analysis software (IPA, Ingenuity ^®^ Systems, www.ingenuity.com, Mountain View, CA, USA) described in Text S1. After 60 min exposure to LeTx, PI3K, insulin and growth factor receptor severely down-regulated (more than two orders of magnitude) in the canonical NF-κB signaling pathway (Fig. S1). There were 20, 7 and 4 fold inductions of *p100, p50* and *p65*, respectively, in both of PLx intox and PLxL intox after a 2 h treatment with PLxL (0.1+0.1+1 µg/ml) but these genes were not induced by PLx treatment (Fig. 5). IRF3 (interferon regulatory factor 3) was slightly induced in PLxL treated intoxication-resistant cells. Recently, LPS-tolerant macrophages were found to have an alternative phenotype in which p50 played an essential role [28].

### Analysis of Jmjd3 and H3K27me3 level by immunofluorescence

**Figure 6:**
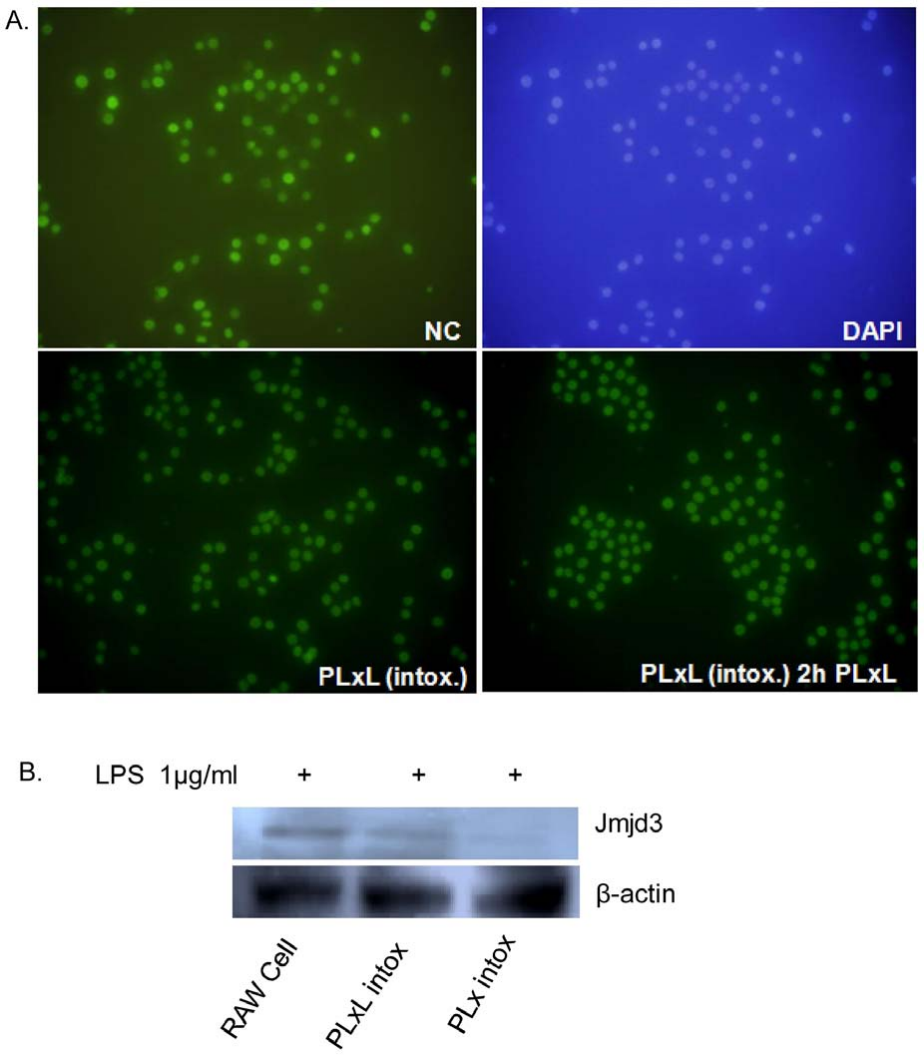
Analysis of H3K27me3 and Jmjd3 expression. A. H3K27me3 was expressed in untreated Raw 264.7 cells, and there was no significant variation in expression in PLxL intoxication-resistant cells (with or without PLxL, 0.1+0.1+1 µg/ml for 2 h treatment). B. Jmjd3 expression was measured by western blotting. Beta-actin was used as a loading control. Here, one of three representative experiments is shown.

Jmjd3 has both conserved iron-binding residues and a conserved α-ketoglutarate (α-KG) binding site, and it shows enzymatic activity, which was assayed using histone 3 as substrate. Jmjd3 demethylated H3K27me3 and, with lower efficiency, H3K27me2 in an iron-dependent manner, showing that Jmjd3 is true histone H3K27me3 demethylase [17]. The global H3K27me3 level in RAW 264.7 and intoxication-resistant cells were measured. Although untreated RAW 264.7 cells (Negative Control/NC) showed better expression, there was no significant variation in the level of H3K27me3 in PLxL intox cells (with or without PLxL, 0.1+0.1+1 µg/ml, 2 h treatment) (Fig. 6A). Although Jmjd3 was found in RAW 264.7 and PLxL intoxication resistant cells clearly with 2 h treatment of LPS (1 µg/ml) (Fig. 6B). Given these results, it is possible that chromatin immunoprecipitation analysis would give a better indication of the presence of H3K27me3 and Jmjd3 at the promoter of a particular gene.

## Discussion

NF-κB activation is induced by a variety of stimuli, including inflammatory cytokines, phorbol esters, bacterial toxins (such as lipopolysaccharides), viruses, UV light and different mitogens. Some co-activator and co-repressor proteins have also been shown to be required for the regulation of gene expression by many transcription factors through altering chromatin structure. Co-activator proteins include CREB-binding protein (CBP) and its structural homolog, p300, which interact with the p65 subunit of NF-κB to enhance its ability to activate transcription [29]. Recent work has shown that the chromatin histone H3-lysine 4 methyltransferase, SET7/9, is a novel co-activator of NF-κB and that depletion of SET7/9 attenuates the transcription of different NF-κB downstream genes, including the histone H3-lysine 27 demethylase, *JMJD3* [30]. These studies suggest that both histone methylase and demethylase might be necessary for NF-κB activity. The demethylase, Jmjd3, was chosen as the subject of this study of intoxication-resistant cells on the basis of two criteria, namely, that it can be regulated by NF-κB and that it is vital for macrophage plasticity. After 60 min of exposure to LeTx, the expression of several key NF-κB signaling genes, chiefly phosphatidylinositol 3-kinase (PI3K) was impaired (Fig. S1). It was reported that the phosphatidylinositol 3-kinase signaling pathway is important in recovery from anthrax toxin-induced cell cycle arrest [11]. LPS tolerant macrophages were characterized by low NF-κB activity and impaired expression of NF-κB dependent genes (e.g. *TNF*, *IL-12* and *IFN*-*β*) [31]. When the expression of NF-κB dependent genes from intoxication-resistant cells was analyzed, a significant induction in the expression of *p100, p50* and *p65* was found in both PLx intox and PLxL intox after 2 h of treatment with PLxL (Fig. 5). The expression of several genes was altered after 180 min of LeTx treatment including dual-specificity phosphatase 6 (*Dusp6*), which was down-regulated, and TNF receptor-associated factor 1 (*Traf1*) and *c-Mer* proto-oncogene tyrosine kinase (*Mertk*), which were up-regulated (data not shown). The effects of these different alterations in gene expression might be to protect macrophages from cell death. It was suggested that resistant cells generated by a 4 h exposure to LeTx exposure might have alternative molecular functions [20].

Intoxication-resistant cells generated in this study were a resistant population of normal macrophages in which the different NF-κB genes, especially *p100, p50* and *p65,* were abundantly expressed. At the same time, the expression of the H3K27me3 demethylase, *Jmjd3*, was also increased. Although NF-κB is required for Jmjd3 expression, the increased expression of Jmjd3 may be crucial to the formation of the open chromatin necessary to change the gene expression profile of intoxication-resistant cells. Accumulation of the p50 homodimer in monocytes/macrophages was described as mediating tolerance to LPS and was found in peripheral blood monocytes of septic patients [28]. The data from this study strongly suggest that increased levels of Jmjd3, in addition to the differential expression of NF-κB genes, are vital for the LPS induced LeTx tolerance of macrophage cells and could be the factors responsible for epigenetic regulation in intoxication-resistant cells.

## Materials and Methods

### Reagents

*Bacillus anthracis* protective antigen (P), lethal factor (Lx), mutant lethal factor (mLF) and lipopolysaccharides (LPS) were from Sigma-Aldrich (USA). RPMI 1640, fetal bovine serum (FBS), and penicillin-streptomycin (PS) were purchased from Gibco Life Technologies (UK). Anti-JMJD3 (AP1022b) was collected from Abgent and anti-rabbit IgG-HRP was collected from Jackson Immuno. Res. Lab. Inc.

### Cell culture

Murine macrophage cell line, RAW 264.7, was originally obtained from the American Type Culture Collection (Manassas, VA). Cell cultures were maintained at sub-confluence at 37°C in a 5% CO_2_ humidified atmosphere using RPMI medium supplemented with 10% fetal bovine serum (FBS), 100 IU/ml penicillin and 10μg/ml streptomycin. One day prior to treatment, cells were seeded at 4×10^6^ cells/100-mm plate (Nunc™, Kamstrupvej, Denmark).

### Cell proliferation assay

Cell proliferation was assayed by a tetrazolium salt colorimetric assay using PreMix WST-1 according to manufacturer's instructions (Takara Bio Inc., Shiga, Japan). Briefly, cells were seeded at a density of 1.5×10^4^ cells per well in 96-well plates in a volume of 100 µl, and they were incubated overnight. PreMix WST-1 was added as indicated and incubated for an additional 4 h, and then the absorbance was read at 450±20 nm. The experiment was performed in triplicate, and the results were analyzed using a Student's *t*-test of three independent experiments.

### Quantitative real time RT-PCR

Primers were designed using Primer Express (Applied Biosystems, Foster City, CA), and all primers are listed in Table 2. Real time RT-PCR was performed using the SYBR Green PCR Master Mix (Takara Bio Inc., Shiga, Japan) and the 7500 fast real time PCR system (Applied Biosystems, Foster City, CA) as were previously described [32]. Briefly, the reaction mixture contained 2 X SYBR^®^ Green PCR Master Mix (Takara Bio Inc., Shiga, Japan), 10 pmol of the forward and reverse primers and 0.05 µg cDNA from treated and untreated RAW 264.7 cells. At each cycle, the accumulation of PCR products was detected by monitoring the increase in fluorescence of the double stranded DNA-binding SYBR Green reporter dye. A typical protocol included a denaturation step at 95°C for 15 min, followed by 40 cycles of 95°C denaturation for 30 sec, 60°C annealing for 1 min and 72°C extension for 1 min. Following the real time RT-PCR, a dissociation curve (melting curve) was generated using a temperature ramp of 60 to 95°C. Glyceraldehyde-3-phosphate dehydrogenase (GAPDH) was used as an internal control. All data were analyzed using the 2^−ΔΔ^CT method [33].

**Table 2:** List of primers used for quantitative real time RT-PCR.

Gene	Forward Primer	Reverse Primer
*Jmjd1a*	5′-CACATTTAGGTTCCCAGTCACA -3	5′-GCCACGATGTTAACACAGGA-3′
*Jmjd1b*	5′-TTCTGCTGGAAGGCTCACTT -3′	5′-GATGCATCCCATTAGCATCC-3′
*Jmjd1c*	5′- AGAAGAGAAAGGCGAGGTC-3′	5′-TTGGGACCTATCTCACAGCA-3′
*Jmjd2a*	5′-GACCACACTCTGCCCACAC-3′	5′- TCCTGGGGTATTTCCAGACA-3′
*Jmjd2b*	5′- GGCTTTAACTGCGCTGAGTC-3′	5′- GTGTGGTCCAGCACTGTGAG-3′
*Jmjd2c*	5′- CACGGAGGACATGGATCTCT-3′	5′- CGAAGGGAATGCCATACTTC-3′
*Jmjd2d*	5′- GTCTTGGTCGTCGTCCTTGT-3′	5′- AATCCCCCTTCAGAAGCTGT-3′
*Jmjd3*	5′ - CCCCCATTTCAGCTGACTAA-3′	5′ – CTGGACCAAGGGGTGTGTT-3′
*Jmjd4*	5′- CTCAAGGACTGGCATCTGTG-3′	5′- CTGAGGAGCGGAAGATGTC-3′
*Jmjd5*	5′-TGTCATGTTAGAGCGGATGG-3′	5′- TGTACCTTGAGCCCACTTCC-3′
*p65*	5′ - AGGCTTCTGGGCCTTATGTG-3′	5′- TGCTTCTCTCGCCAGGAATAC-3′
*p50*	5′ - GGAGGCATGTTCGGTAGTGG-3′	5′- CCCTGCGTTGGATTTCGTG-3′
*p100*	5′ - GGCCGGAAGACCTATCCTACT-3′	5′- CTACAGACACAGCGCACACT-3′
*IRF3:*	5′- GAGAGCCGAACGAGGTTCAG-3	5′ - CTTCCAGGTTGACACGTCCG-3′

### Western blotting

Western blotting was performed following standard procedures [34]. Briefly, equal amounts of protein from nuclear and cytoplasmic extracts (20 µg) were resolved by electrophoresis on 10% or 12% polyacrylamide gels for Jmjd3 or β-actin, respectively. Proteins were then transferred to polyvinylidene difluoride (PVDF) membranes (Schleicher & Schuell Bioscience, Inc., Keene, NH) by electroblotting using the immersion method. The membranes were blocked with 5% skimmed milk in 1% TBS-Tween for one hour and incubated overnight with primary antibodies at 4°C. After washing, the membranes were incubated for one hour with the secondary antibody. Blots were visualized with enhanced chemiluminescence (ECL™ Plus Western Blotting Detection Kit; GE Healthcare, Piscataway, NJ).

### Immunofluorescence analysis

Cells were fixed for 30 min at room temperature with 4% (w/v) paraformaldehyde in phosphate buffered saline (PBS) and were then permeabilized with 0.3% (v/v) Triton X-100 in PBS. Fixed cells were incubated overnight with the indicated primary antibodies.

### Statistical analysis

All values are expressed as the mean± standard deviation (SD). Statistical analysis was performed using SPSS 17.0 (SPSS Inc., IL, USA). Data were tested using a one-way ANOVA followed by the Tukey's HSD *post hoc* test. *P*-value of <0.05 were considered significant.

## Supporting Information

Figure S1 Effect of LeTx on RAW 264.7 cells after 60 min, 90 min and 180 min. IPA analysis was performed. Exposure to LeTx (60 min) showed severe down-regulation (more than two orders of magnitude) of PI3K, insulin and growth factor receptor in the canonical NF-κB signaling pathway. The molecules colored white were not affected by LeTx. The molecules colored green were down-regulated in response to LeTx. The node color indicates the expression level of the genes and the brightness of node colors is proportional to the fold changes of gene expression levels.
(0.41 MB TIF)

Text S1 (0.03 MB DOC)
