# Supplementary figures and images for: The Role of NF-κB and H3K27me3 Demethylase, Jmjd3, on the Anthrax Lethal Toxin Tolerance of RAW 264.7 Cells
Source: PLoS One. 2010 Mar 29;5(3):e9913. doi: 10.1371/journal.pone.0009913 (PMC2848010; doi:10.1371/journal.pone.0009913)

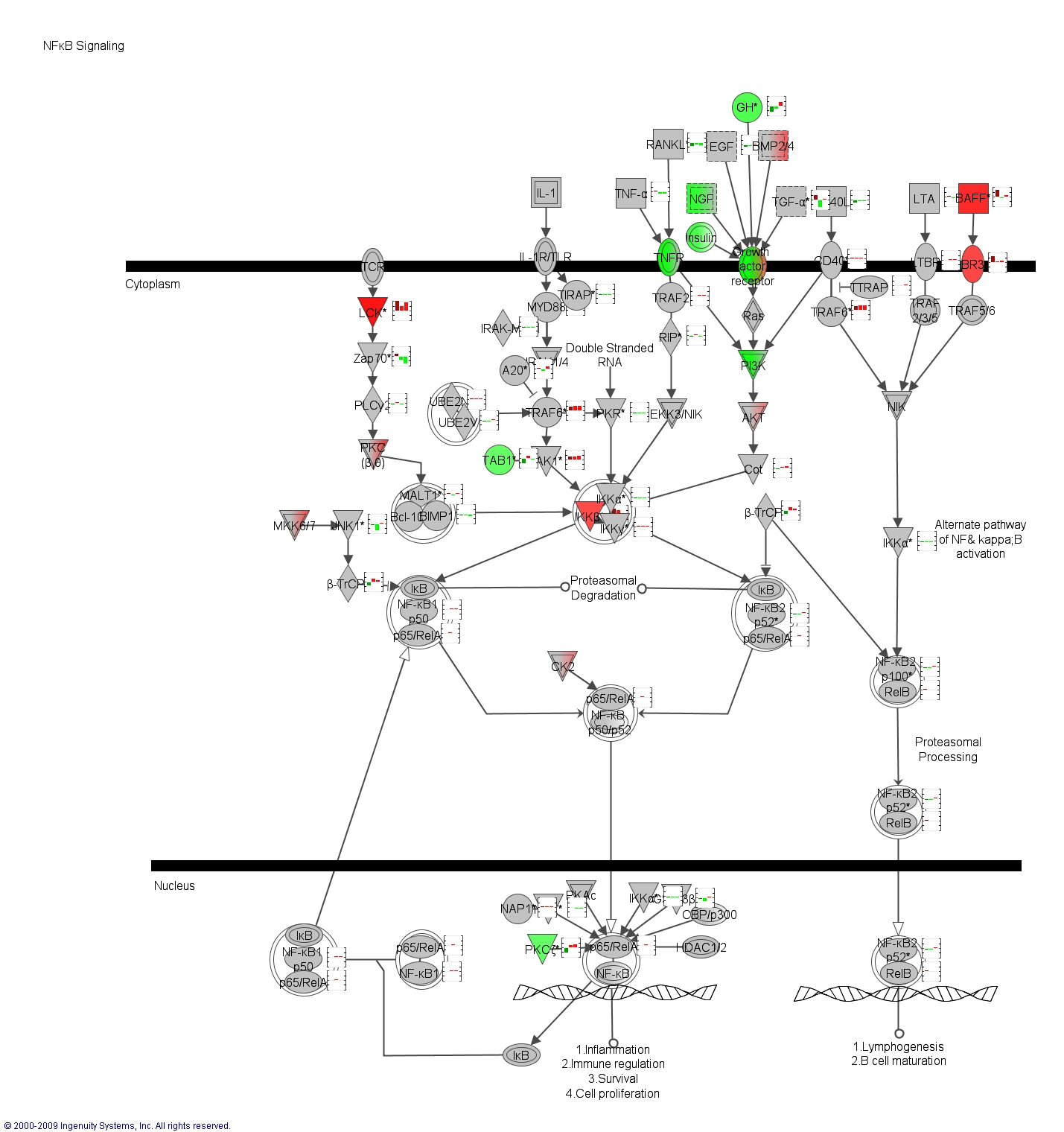

Supplement: Figure S1 — Effect of LeTx on RAW 264.7 cells after 60 min, 90 min and 180 min. IPA analysis was performed. Exposure to LeTx (60 min) showed severe down-regulation (more than two orders of magnitude) of PI3K, insulin and growth factor receptor in the canonical NF-κB signaling pathway. The molecules colored white were not affected by LeTx. The molecules colored green were down-regulated in response to LeTx. The node color indicates the expression level of the genes and the brightness of node colors is proportional to the fold changes of gene expression levels. (0.41 MB TIF) [file pone.0009913.s001.tif]
