# Supplementary material for: The Role of NF-κB and H3K27me3 Demethylase, Jmjd3, on the Anthrax Lethal Toxin Tolerance of RAW 264.7 Cells
Source: PLoS One. 2010 Mar 29;5(3):e9913. doi: 10.1371/journal.pone.0009913 (PMC2848010; doi:10.1371/journal.pone.0009913)
Supplement: Text S1 — (0.03 MB DOC) [file pone.0009913.s002.doc]

**Canonical pathway analysis of datasets**

Ingenuity pathway analysis (IPA, Ingenuity ® Systems, www.ingenuity.com, Mountain View, CA, USA) was conducted to analyze the different canonical pathways that were most significant in the datasets. Briefly, genes from the dataset that were associated with a canonical pathway in the Ingenuity Pathways Knowledge Base were considered for the literary analysis. The significance of the association between the dataset and the canonical pathway was measured in two ways: (1) the ratio of the number of genes from the dataset that map to a given canonical pathway divided by the total number of genes that map to the same canonical pathway was determined. (2) Fischer’s exact test was used to calculate a p value determining the probability that the association between the genes in the dataset and the canonical pathway could be explained by chance alone. After uploading the datasets, each gene identifier was mapped to its corresponding gene object in the Ingenuity Pathways Knowledge Base, and these genes were overlaid onto a global molecular network developed from information contained in the Ingenuity Pathways Knowledge Base. Networks of these genes were then algorithmically generated based on their connectivity.

**Graphical representation of networks/pathways**

The graphical representation of molecular relationships between genes/gene products were based on the following. Genes or gene products are represented as nodes, and the biological relationship between two nodes is represented as an edge (line). All edges are supported by at least one reference from the literature, from a textbook or from canonical information stored in the Ingenuity Pathways Knowledge Base. The intensity of the node color indicates the degree of up- (red) or down- (green) regulation. Nodes are displayed using various shapes that represent the functional class of the gene product (Δ-phosphatase, ◊-enzyme, ▼-kinases and О-others).
